# Glial Cells in Behavioral and Psychological Symptoms of Alzheimer’s Disease

**DOI:** 10.3390/ijms27104621

**Published:** 2026-05-21

**Authors:** Ilminur Hasan, Xiaoyu Tang, Jianrong Xu

**Affiliations:** School of Integrative Medicine, State Key Laboratory of Discovery and Utilization of Functional Components in Traditional Chinese Medicine and Shuguang Hospital, Shanghai University of Traditional Chinese Medicine, Shanghai 201203, China; 22024045@shutcm.edu.cn (I.H.); 13918575220@163.com (X.T.)

**Keywords:** Alzheimer’s disease, behavioral and psychological symptoms of dementia, glial cells, microglia, astrocytes, oligodendrocytes

## Abstract

Behavioral and psychological symptoms of dementia (BPSD) affect the majority of patients with Alzheimer’s disease (AD), substantially increasing caregiver burden and the likelihood of institutionalization. The clinical management of BPSD remains challenging because of its poorly understood pathogenesis, the limited efficacy of conventional interventions, and significant safety concerns associated with current treatments. These limitations underscore the urgent need to identify novel therapeutic targets and develop glia-centered treatment strategies. As essential components of the central nervous system, glial cells maintain neural homeostasis, regulate neurotransmission, and mediate neuroinflammatory responses. Increasing evidence suggests that glial dysfunction contributes to the development of BPSD, thereby linking AD neuropathology and neuropsychiatric symptoms. Aberrant microglial activation, astrocytic dysfunction, and oligodendrocyte injury collectively compromise neural circuit integrity, disrupt neurotransmitter balance, and impair neuron–glia communication, ultimately promoting the progression of diverse BPSDs. Given the critical role of glial cells in regulating neurotransmitter systems, the dysregulation of which is closely associated with BPSD, this review summarizes the involvement of glial cells in BPSD, elucidates the underlying molecular mechanisms, and discusses recent advances in glia-based therapeutic strategies, thereby providing insights into the pathogenesis of BPSD in AD.

## 1. Introduction

BPSD encompasses a heterogeneous spectrum of neuropsychiatric manifestations, including mood disturbances, apathy, agitation, and psychosis. In AD, more than 90% of patients experience these debilitating symptoms during the course of the disease [1]. BPSD is a major contributor to patient distress, caregiver burden, and institutionalization—in fact, it is the leading cause of early nursing home placement, even more so than core cognitive deficits, yet effective therapeutic strategies are still lacking [2]. The clinical significance of BPSD is further supported by recent meta-analyses demonstrating that approximately 60% of hospitalized patients with dementia exhibit BPSD, among which aggression, sleep disturbances, and irritability are the most prevalent symptoms. These manifestations are associated with poor clinical outcomes and frequent hospital readmissions [3]. In addition, social isolation, a well-established risk factor for depression, substantially exacerbates mental health deterioration in cognitively vulnerable older adults. During COVID-19-related social isolation, nearly one-third of cognitively healthy older individuals, and an even higher proportion of those with MCI or dementia, reported worsening mental health status, with depression being the most commonly reported symptom [4].

Clinically, BPSD domains frequently overlap with cognitive decline and lack objective biomarkers, relying primarily on subjective behavioral assessment scales that inadequately capture the underlying biological heterogeneity [5]. Current pharmacological treatments, such as antipsychotics, antidepressants, and cholinesterase inhibitors, provide limited therapeutic benefit and are associated with significant safety concerns, thereby underscoring the urgent need for mechanism-based therapeutic strategies. Although the pathogenesis of AD has traditionally focused on Aβ and tau pathology, glial cells—particularly microglia, astrocytes, and oligodendrocytes—are now recognized as active contributors to the disease progression. Dysfunction of these cells is characterized by chronic activation, the release of inflammatory cytokines, and loss of homeostatic support, thereby promoting neuroinflammation, synaptic impairment, and excitotoxicity [6,7,8,9]. Nevertheless, most existing glia-focused studies have primarily focused on cognitive impairment, while the role of glial cells in contributing to BPSD is still poorly defined.

Emerging evidence suggests that glial mechanisms may directly contribute to specific BPSD domains beyond cognitive impairment. Transcriptomic analyses have identified distinct gene expression signatures associated with different BPSD clusters, implicating glial-regulated pathways, such as extracellular matrix remodeling [8,9]. Furthermore, glial cells serve as key regulators of neurotransmitter systems and stress responses, both closely associated with mood disturbances, anxiety, aggression, and psychosis. Accordingly, BPSD may be conceptualized as a glia-driven circuit disorder in which aberrant glial activity disrupts neural circuits involved in emotional and behavioral regulation.

**Figure 1:**
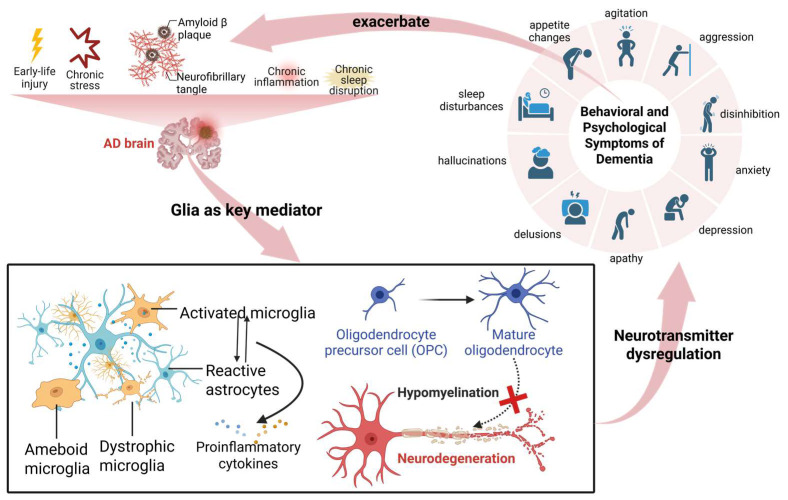
Glial cells as key mediators in BPSD of AD. Created in BioRender. Hawthorn, M. (2026) https://BioRender.com/d2a4v8b (accessed on 17 May 2026).

However, the roles of distinct glial subtypes and their interactions in driving specific BPSD domains remain poorly understood. In this review, we synthesize current evidence on glial pathophysiology in AD-related BPSD, focusing on glia-mediated neurotransmitter regulation, symptom-specific pathological mechanisms, core molecular pathways, and emerging glia-based therapeutic strategies. Figure 1 presents a concise schematic overview of the pivotal contributions of glial cells to BPSD pathogenesis. This review is intended for a broad audience, including basic neuroscientists interested in glia–neuron crosstalk, drug discovery researchers seeking glia-targeted therapies, and clinical investigators aiming to translate mechanistic insights into BPSD management.

## 2. Glia as Key Mediators of BPSD of AD

### 2.1. AD and Dementia

AD is the most prevalent neurodegenerative disorder and the leading cause of dementia worldwide, accounting for approximately 60–80% of all dementia cases. Current estimates indicate that nearly 50 million individuals are living with AD globally, and this number is projected to increase to 152 million by 2050, imposing an annual economic burden of approximately $1 trillion [10,11]. Although AD predominantly occurs after the age of 65, early-onset forms of the disease can also affect younger individuals. Clinically, AD is characterized by progressive memory decline, aphasia, agnosia, visuospatial dysfunction, executive impairment, as well as prominent personality and behavioral changes [12,13].

At the neuropathological level, AD is characterized by three core pathological features: extracellular Aβ plaques, intracellular neurofibrillary tangles composed of hyperphosphorylated tau, and chronic neuroinflammation [13]. Neuroinflammation is primarily mediated by glial cells, particularly microglia and astrocytes, which become reactive in response to proteinopathies. Clinically, AD follows a progressive trajectory beginning with a preclinical phase characterized by silent Aβ accumulation lasting 10–20 years, followed by a prodromal stage of MCI, and ultimately progression to dementia [14,15]. Notably, although memory impairment represents the hallmark feature of early AD, neuropsychiatric and behavioral symptoms may emerge throughout the disease course, often accelerating functional deterioration and contributing to institutionalization [16]. The concurrent emergence of glial activation and behavioral symptoms in AD suggests that glial cells may directly contribute to BPSD, extending beyond their well-established involvement in cognitive impairment.

### 2.2. BPSD in AD

**Figure 2:**
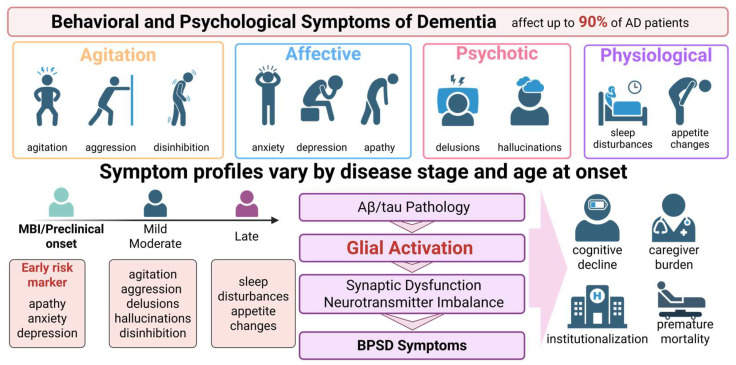
Clinical manifestations and symptom classification of BPSD in AD. Created in BioRender. Hawthorn, M. (2026) https://BioRender.com/d2a4v8b (accessed on 17 May 2026).

AD is the most common neuropathological substrate of BPSD. A systematic review demonstrated that ADNC accounts 83.44% of 18,823 cases presenting with hyperactive or psychotic symptoms [17]. Therefore, AD serves as an important model for elucidating the mechanisms underlying BPSD, including the contribution of glial cells. BPSD encompasses a heterogeneous spectrum of non-cognitive symptoms, including agitation, aggression, anxiety, depression, apathy, delusions, hallucinations, disinhibition, as well as disturbances in sleep and appetite [18]. In AD, BPSD affects up to 90% of patients [19] and is associated with accelerated cognitive deterioration, institutionalization, premature mortality, and increased caregiver burden [20]. These symptoms constitute a major component of disease burden, often more distressing than cognitive deficits themselves [21], yet they remain underrecognized and are frequently regarded as inevitable consequences of dementia [22]. BPSD exhibits substantial clinical heterogeneity. Factor-analytic studies have identified relatively stable symptom subsyndromes, including affective, agitation/aggression, and psychosis [23], although their clinical presentation varies considerably among individuals and across disease stages [24]. Age at disease onset also influences BPSD profiles. Compared with early-onset AD, late-onset AD is associated with more severe delusions, agitation, anxiety, disinhibition, and nighttime disturbances [25]. Moreover, BPSD manifestations vary across the AD continuum, with certain symptoms emerging during prodromal stages, even before marked cognitive impairment becomes evident [5]. MBI has therefore been proposed as an early at-risk state, in which apathy and affective symptoms may arise during preclinical/prodromal phases [5,26]. Notably, apathy in patients with MCI significantly increases the risk of conversion to AD dementia (HR = 1.54) [27]. The severity and frequency of BPSD generally increase with disease progression and typically peak during the moderate stages of AD [28]. The clinical manifestations, classification and proposed mechanistic cascades of BPSD in AD are summarized in Figure 2.

Despite the substantial clinical significance of BPSD, its pathophysiological mechanisms remain incompletely understood. Accumulating evidence suggests that BPSD arises from complex interactions among anatomical, functional, genetic, and biochemical alterations [24]. Notably, disruptions in neuron–glia crosstalk and neuroimmune communication have been observed from the earliest stages of prodromal AD, during which early BPSDs may already emerge while cognitive function remains relatively preserved [26]. These findings raise the possibility that clinical heterogeneity reflects underlying neurobiological diversity, including variations in glia-related pathways. Intriguingly, behavioral and psychological symptoms may precede clinical AD diagnosis by decades. For example, depressive symptoms in midlife, particularly concentration difficulties, have been associated with an increased risk of dementia more than 20 years later [29]. Furthermore, psychiatric multimorbidity, including depression, anxiety, and schizophrenia) is associated with a substantially greater risk of dementia (HR = 2.81) compared with single psychiatric disorders, suggesting that coexisting psychiatric conditions may accelerate neurodegeneration, potentially through sustained neuroglial dysfunction [30]. Collectively, these clinical observations support the notion that the heterogeneity of BPSD may reflect distinct neurobiological trajectories in which glial cells serve as key mediators.

### 2.3. Glial Involvement in BPSD of AD

A growing body of evidence identifies glial cells as critical intermediaries linking AD pathology to behavioral manifestations. Genetic studies provide preliminary, albeit indirect, evidence supporting this association. The APOE ε4 allele—expressed predominantly in astrocytes and microglia—is significantly associated with psychosis and hyperactivity endophenotypes, whereas variants of MTHFR, which influence glial inflammatory states, are linked to delusions [31]. These findings suggest that genetic susceptibility to BPSD may be mediated, at least in part, through glia-related pathways, thereby translating genetic risk into neural circuit dysfunction. Beyond genetic evidence, findings from animal models further support the involvement of glial cells in BPSD. In 3xTg-AD mice, stage-dependent microglial transitions, ranging from early immunosuppression to overt neuroinflammation by 9 months of age, parallel the emergence of behavioral symptoms [32]. A bioinformatics study conducted by Zhang et al. (2025) further demonstrated that neuroinflammation links early-life brain injury to AD-associated behavioral deficits, with dysregulated glial signaling pathways, including AKT1 and MAPK14, contributing to anxiety-like behaviors [33]. Moreover, in a mouse model co-expressing tau, Aβ, and α-synuclein, microglial activation and peripheral immune infiltration were synergistically enhanced compared to single pathology conditions, suggesting that mixed pathologies drive a more robust glial response that may underlie severe or rapidly progressive BPSD [34]. Perhaps most compellingly, human neuroimaging studies have established a direct association between microglial activation and BPSD severity. Using TSPO-PET ([^11^C]PBR28) in 109 individuals across the AD continuum, a study demonstrated that higher microglial activation was significantly associated with greater neuropsychiatric symptom severity (β = 7.37), independent of Aβ and tau pathology [35]. Another PET study further showed that the severity of irritability correlates with cortical microglial activation independently of AD proteinopathies [36]. Collectively, these fundings support an independent contribution of neuroinflammation to BPSD, beyond classical protein aggregation.

Emerging evidence further indicates that glial cells disrupt neural network communication through pro-inflammatory pathways such as NF-κB signaling, thereby leading to neuronal dysfunction and circuit dysregulation [37]. In the prefrontal cortex, glia-driven neuroinflammation and excitotoxicity correlate with neuropsychiatric symptoms [38]. Specifically, reactive glial cells impair synaptic transmission and neurotransmitter homeostasis, and reduced synaptic density is linked to affective disturbances [39]. Notably, glia-induced synaptic dysfunction modulates emotion-related circuits in a region-specific manner, with amygdala hyperactivity driving agitation and prefrontal hypoactivity underlying apathy, thereby providing a circuit-based framework for symptom-specific BPSD. Moreover, glial dysfunction exhibits marked regional heterogeneity, including differences between prefrontal and limbic regions, and stage-dependent alterations, thereby explaining the dynamic and variable presentation of BPSD [36,40].

Taken together, the traditional neuron-centric view of AD has been broadened to recognize glial cells as active contributors to both neuropathology and behavioral symptoms. Future studies employing region- and cell-type-specific models (e.g., brain organoids incorporating human microglia) are warranted to establish direct links between specific glial dysfunctions and distinct BPSD subsyndromes [41].

## 3. Pathophysiology of Glial Cells in BPSD

**Figure 3:**
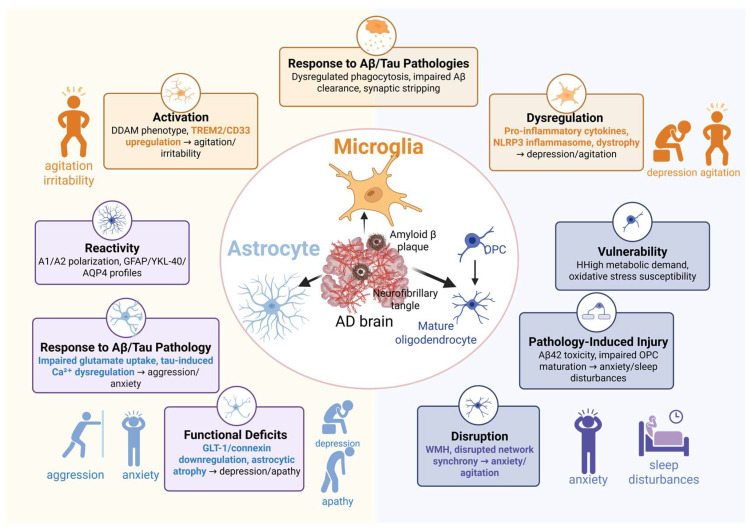
Spectrum of glia-mediated mechanisms linking AD pathology to the BPSDs. Color coding: orange, microglia-related mechanisms; purple, astrocyte-related mechanisms; blue, oligodendrocyte/OPC-related mechanisms. Arrows indicate the causal sequence: AD pathologies (Aβ plaques and neurofibrillary tangles) lead to glial dysfunction and subsequent BPSD manifestations. Created in BioRender. Hawthorn, M. (2026) https://BioRender.com/d2a4v8b (accessed on 17 May 2026).

The role of glial cells in the pathogenesis of BPSD has received increasing attention in recent years. The specific mechanisms through which glial cells mediate the relationship between AD pathology and BPSD are illustrated in Figure 3, which summarizes the distinct contributions of different glial cell types in driving heterogeneous BPSDs.

### 3.1. Microglia

Microglia, the resident immune cells of the central nervous system, have traditionally been investigated in the context of neurodegeneration and cognitive impairment. However, accumulating evidence now implicates microglial dysfunction in the development of BPSD.

Microglial Activation and Clinical Correlates. Resting microglia undergo a transition to an activated state, characterized by morphological changes, upregulation of surface markers, including TREM2, CD33, and CR3, as well as increased cellular proliferation. Spatial transcriptomic analyses have revealed that microglia surrounding Aβ plaques acquire a distinct DAM phenotype characterized by the expression of Trem2, Apoe, Clec7a, and Cst7. This phenotype is particularly enriched within 10–20 µm of plaques, where these microglia orchestrate local synaptic stripping and neuroinflammation that may contribute to neural circuit dysfunction in BPSD [42]. In AD, this transition from a homeostatic state to a DAM phenotype is driven by signaling pathways, such as the TREM2-APOE axis, and involves TREM2-dependent downregulation of homeostatic genes, including *P2RY12*, with concomitant upregulation of DAM-associated markers such as APOE and SPP1. During the early stages of AD, DAM microglia may exert protective effects by forming a barrier around Aβ plaques and facilitating plaque clearance. However, during later stages, chronic microglial activation leads to sustained release of pro-inflammatory cytokines, including IL-1β and TNF-α, as well as aberrant complement-mediated synaptic pruning. These pathological processes have been directly linked to neuronal injury and cognitive decline. This dual role highlights how microglial dysfunction, particularly the transition toward a pro-inflammatory, neurotoxic phenotype, may contribute to the neural circuit disruptions underlying BPSD, such as agitation, depression, and apathy [43].

Microglial polarization between pro-inflammatory (M1-like) and anti-inflammatory (M2-like) phenotypes plays a critical role in maintaining neuronal homeostasis, and the promotion of M2-like states has been shown to confer resilience to depressive-like behaviors [44]. Human neuroimaging studies have further established direct associations between microglial activation and specific BPSDs. A TSPO-PET study published in 2023 demonstrated that microglial activation was associated with agitation and frontal symptoms in patients with AD, independent of cognitive status and proteinopathy [35]. In a leave-one-out analysis, irritability was identified as the neuropsychiatric domain most strongly associated with brain microglial activation (β = 6.86; 95% CI, 1.77–11.95; *p* = 0.008), followed by nighttime disturbances and agitation. Region-specific analyses revealed that this microglia-BPSD association was prominent in the posterior cingulate, precuneus, inferior temporal, and anterior cingulate cortices—areas involved in default mode and salience networks [35]. Thus, these findings suggest that neuroinflammation in specific circuits underlies particular behavioral symptoms.

Microglial Response to Aβ/Tau. Microglia drive BPSD by impairing protein aggregate clearance and aberrant synaptic pruning, with age-related receptor changes exacerbating these dysfunctions. Specifically, upregulation of TREM2 enhances Aβ clearance but may exacerbate chronic inflammation, while elevated CD33 suppresses phagocytosis and promotes Aβ accumulation [45]. A study published in 2025 further demonstrated that neuronal CD47, a “don’t eat me” signal, inhibits microglial synaptic phagocytosis; CD47 overexpression in AD models alleviated excessive synaptic loss, suggesting a context-dependent relationship between pruning and behavior [46]. Whether aberrant reactivation of developmental pruning programs in dementia recapitulates development or represents a distinct process remains an open question.

Microglial Dysregulation. Activated microglia release IL-1β, TNF-α, and IL-6, which disrupt serotonergic transmission by modulating SERT, thereby contributing to neuropsychiatric symptoms [47]. The NLRP3 inflammasome and the complement cascade have also been implicated. A 2018 review identified microglial pro-inflammatory activity as a central contributor to behavioral disturbances in AD, noting that both cognitive and non-cognitive symptoms—including apathy, depression, aggression, sleep disorders—converge on shared neuroinflammatory pathways [48]. The histaminergic system represents an additional link between microglial function and BPSD. However, most studies treat BPSD as a secondary outcome; yet the dissociation between cognitive and behavioral symptoms implies the existence of partially distinct pathways, with microglial activation differentially affecting prefrontal-limbic circuits as opposed to hippocampal circuits. Genetic modifiers play a significant role. For instance, TREM2 regulates phagocytosis, survival, and lipid metabolism; its R47H variant triples the risk of AD. TREM2-deficient mice exhibit altered social behavior, excessive synaptic retention, and anxiety-like/repetitive behaviors all of which have been attributed to impaired pruning [49]. In humans, CSF sTREM2 correlates with blood–brain barrier dysfunction; this finding is relevant given that depression—which has a prevalence of 42% in AD—may involve suppressed microglial activation [50,51]. Other variants, including CD33, CR1, and APOE, also modulate microglial function, although their links to BPSD remain less explored.

Beyond genetic risk, microglial phenotypic heterogeneity—spanning from homeostasis to dystrophy—directly contributes to BPSD. Human postmortem studies reveal a spectrum: homeostatic ramified cells, reactive amoeboid forms, and dystrophic or senescent cells characterized by fragmented processes and reduced phagocytic capacity [52,53]. Microglial dystrophy is prominent in AD brains and precedes neurofibrillary degeneration [52]. The shift from protective to harmful phenotypes is driven by aging, oxidative stress, and interactions with Aβ and phosphorylated tau. For instance, Aβ accelerates microglial senescence, as evidenced by the upregulation of p21 and beta-galactosidase activity [54]. Specific reactive states, such as DAM or LDAM are associated with the release of pro-inflammatory cytokines (IL-1β, TNF-α) and impaired debris clearance [55,56]. Given that neuroinflammation and synaptic dysfunction are implicated in BPSD, this microglial heterogeneity—ranging from exhausted phagocytic capacity to neurotoxic overactivation—provides a direct cellular mechanism linking AD pathology to symptoms such as agitation, depression, and apathy.

Microglia are not passive responders but rather active contributors to BPSD, acting through diverse mechanisms including activation heterogeneity, synaptic pruning dysregulation, release of inflammatory mediators, genetic susceptibility, and phenotypic state transitions. However, most current evidence remains correlational; future studies employing longitudinal designs and microglia-specific interventions are warranted to establish causality and identify symptom-specific therapeutic targets.

### 3.2. Astrocytes

Astrocytes, once viewed as passive supportive cells, are now recognized as active regulators of neural circuits underlying behavior, and their dysfunction is increasingly implicated in BPSD.

Astrocyte Reactivity. Astrocyte reactivity—characterized by morphological, molecular, and functional changes—is increasingly linked to BPSD. Using human AD and PD cohorts, Li et al. (2025) identified distinct CSF astrocyte protein clusters (GFAP, YKL-40, AQP4): the “highYKL|lowOthers” cluster was associated with lower severity of hallucinations, anxiety, disinhibition, and sleep disturbances [57]. Rodent models of depression have demonstrated that altered astrocyte-neuron crosstalk produces behaviors resembling human NPS [58]. Astrocyte responses are heterogeneous, spanning from neurotoxic (A1-like) to neuroprotective (A2-like) phenotypes; this heterogeneity precludes simple conclusions but suggests that modulating astrocyte reactivity toward a protective state could represent a viable therapeutic strategy for BPSD.

Astrocytic Response to Aβ/Tau. Astrocytes actively respond to amyloid and tau pathologies, thereby mediating the cascade from proteinopathy to neuronal network dysfunction. Aβ reduces astrocytic glutamate uptake while increasing glutamate release, leading to neuronal hyperexcitability [59,60]; consistent with this, Aβ1-42-injected mice exhibit increased aggression and anxiety, which are associated with heightened hippocampal glutamatergic excitability [61]. Tau oligomers accumulate in astrocytes, disrupting Ca^2+^ signaling and gliotransmitter release [62]. In tauopathy mice, reactive astrocytes upregulate α2-NKA; its inhibition suppresses astrogliosis, reduces TNF-α and IL-6 levels, and improves nesting behavior (a surrogate measure of apathy) [63]. Moreover, astrocytic Lcn2 exacerbates tau pathology and promotes tau uptake, thereby directly linking astrocytic tau responses to behavioral deficits [63]. Collectively, these findings indicate that astrocytic responses to Aβ and tau actively drive the hyperexcitability underlying agitation and aggression.

Astrocyte-Mediated Synapse Elimination. Beyond aberrant excitability, astrocytes critically mediate synapse elimination—a fundamental process for circuit refinement that becomes dysregulated in neurodegeneration. Using a sensory deprivation model of synaptic remodeling, microglia signal to astrocytes via Wnt ligands (e.g., WNT7A, WNT2B, WNT4) to induce retraction of astrocyte processes from thalamocortical synapses. This retraction, as quantified by an increase in the average synapse-astrocyte nearest neighbor distance from ~0.15 μm to ~0.25 μm and a concomitant decrease in the percentage of synapses contacted by astrocytes, precedes and permits microglia-mediated synapse engulfment [64]. This CX3CL1-CX3CR1-dependent microglia-astrocyte crosstalk highlights a coordinated glial mechanism for activity-dependent synapse removal. In AD, chronic neuroinflammation and aberrant signaling may hijack such physiological pathways, leading to excessive retraction of astrocyte processes and pathological synapse loss. Such alterations could disrupt neural circuits governing mood and motivation, potentially contributing to BPSDs such as apathy and depression. Therefore, Wnt-mediated astrocyte morphological plasticity represents a potential link between glial dysfunction and the synaptic deficits that underlie behavioral disturbances in dementia.

Astrocytic Deficiency. Beyond reactivity, astrocytes exhibit specific functional and structural deficits that directly contribute to BPSD. In pre-plaque AD mice, reactive astrocytes exhibit an elevated tonic GABA current, which induces hippocampal synaptic mistuning and depressive-like behavior [65]. Additionally, hippocampal astrocytes exhibit a loss of learning-induced structural plasticity; specifically, contextual fear conditioning fails to trigger perisynaptic astrocytic process retraction, a deficit that impairs memory consolidation and likely underlies apathy or depression in BPSD [66]. These functional (aberrant GABA signaling) and structural (impaired PAP plasticity) deficits converge to disrupt circuit tuning. Moreover, morphological and molecular evidence further support the role of astrocytic dysfunction in BPSD. In both depression and AD, postmortem and imaging studies have revealed astrocyte atrophy in overlapping brain regions, including the hippocampus and prefrontal cortex [67,68,69]. Consistent with these findings, experimental models of chronic stress, which precipitate depressive symptoms, recapitulate this astrocyte atrophy and can even induce pyroptotic death of hippocampal astrocytes via NLRP3 inflammasome activation [70]. Functionally, these structural changes are accompanied by altered expression of key astrocytic proteins, including reduced levels of glutamate transporters (e.g., GLT-1) and connexins. These alterations impair synaptic glutamate clearance and inter-astrocytic communication, which may underlie both affective and cognitive symptoms in AD [71,72]. Therefore, strategies aimed at restoring astrocytic function—such as correcting ionic imbalances, rescuing structural plasticity, or targeting specific molecular pathways—hold promise for mitigating early synaptic and behavioral deficits in AD [65,66].

In summary, astrocytes are not passive bystanders but active drivers of BPSD, contributing through reactivity to proteinopathies, synapse elimination, neurotransmitter dysregulation, and structural atrophy. However, the field faces two major challenges: first, distinguishing protective reactive astrogliosis from pathogenic responses; and second, developing astrocyte-targeted interventions that modulate specific signaling pathways without compromising homeostatic functions. Addressing these challenges will be critical for translating astrocyte biology into effective BPSD therapeutics.

### 3.3. Oligodendrocyte

Oligodendrocytes, the myelinating cells of the central nervous system, have traditionally been studied in relation to white matter integrity and cognitive function. However, a growing body of evidence now implicates oligodendrocyte dysfunction in the development of BPSD.

Oligodendrocyte Vulnerability. Oligodendrocytes are among the most vulnerable cell types in the aging brain due to their high metabolic demand and limited antioxidant capacity, rendering them particularly susceptible to oxidative stress—a hallmark of AD that accumulates prior to clinical onset [73]. This vulnerability predisposes them to dysfunction under neuroinflammatory and proteinopathic conditions, thereby setting the stage for white matter damage and behavioral disturbances.

Oligodendrocyte Injury by Aβ/Tau. In AD, oligodendrocytes actively respond to amyloid and tau pathologies. Notably, they can produce highly toxic Aβ42 aggregates, while neuron-derived Aβ at high concentrations directly induces oxidative stress and oligodendrocyte apoptosis, thereby disrupting differentiation and myelin production [74]. Tau oligomers may also accumulate within oligodendrocytes, though the mechanisms underlying this accumulation remain poorly understood. These responses directly link proteinopathy to oligodendrocyte injury and subsequent myelin breakdown.

Oligodendrocyte Disruption. A 2025 review has confirmed that myelin disruption underlies cognitive, motor, and behavioral deficits across multiple neuropsychiatric disorders, including schizophrenia, depression, bipolar disorder, and AD [75]. Experimental models provide causal evidence: oligodendrocyte dysfunction impairs synaptic transmission and triggers anxiety, depression, and social abnormalities [76]. In a social isolation mouse model, promoting oligodendrocyte differentiation and myelination with clemastine rescued social avoidance behavior via an H3K9me3 epigenetic mechanism [77]. Consequently, myelin disruption impairs the timing of neural communication and network synchrony, thereby contributing to BPSD. Clinically, MRI WMH—markers of myelin and axonal damage—are strongly and independently associated with BPSD. In a cohort of 122 patients with moderate-to-severe AD, those with BPSD exhibited a significantly higher total WMH burden (*p* < 0.001; OR = 1.45) [78]. Disconnection of frontotemporal and limbic white matter tracts leads to apathy and disinhibition [79]. Importantly, the association between WMH and BPSD persists after adjustment for cognitive decline, suggesting the involvement of mechanisms distinct from those underlying cognitive impairment. Moreover, in AD patients, WMH are specifically associated with anxiety, aberrant motor behavior, and nighttime disturbances [80]. Bartzokis has proposed a unifying model: optimal brain function relies on the synchronization of action potentials enabled by myelin, which represents the CNS’s “weakest link” and is particularly vulnerable to metabolic and oxidative insults. Both developmental myelination deficits and degenerative myelin breakdown can disrupt network synchrony, leading to similar clusters of behavioral symptoms—such as psychosis, depression, and agitation—despite differing etiologies [81]. Collectively, these findings position oligodendrocyte dysfunction and myelin pathology as a critical substrate for BPSD.

### 3.4. Glial Cells Interactions in BPSD

Glial cells do not operate in isolation; their dynamic crosstalk—via cytokines, complement, metabolic signals, and oxidative stress—shapes BPSD-related neural dysfunction.

Cytokine-Mediated Crosstalk. A well-established mechanism involves microglia-driven disruption of serotonergic signaling. AβOs induce depressive-like behavior in mice by binding to microglial TLR4, triggering TNF-α release, and leading to a significant decrease in brain 5-HT levels; conversely, serotonin acts as a negative regulator of microglial activation, as 5-HT treatment prevents AβO-induced microglial activation and TNF-α elevation [82]. Systemic inflammatory challenge induces microglial and astrocytic activation largely mediated by IL-1β, creating a positive feedback loop that further elevates IL-1β, COX-2, and PGE2. Blockade of this loop by IL-1ra reduces glial activation and ameliorates associated behavioral hypersensitivity [83]. Additionally, activated microglia can drive astrocytes toward a detrimental, neuroinflammatory A1 phenotype via the release of IL-1α, TNF-α, and C1q [84], whereas astrocytes can modulate microglial activity through the release of GDNF [85].

Complement System and Synaptic Pruning. The complement system mediates microglial phagocytosis, and its dysregulation disrupts neural circuit integrity. CR3 deficiency in mice impaired neuronal clearance in the anterior cingulate cortex during early development, resulting in increased neuronal density and enhanced local functional connectivity in adulthood [86]. Mice lacking the microglial fractalkine receptor CX3CR1 exhibit defective developmental synaptic pruning, reduced synaptic density, diminished prefrontal-hippocampal functional connectivity, and BPSD-like behaviors, including decreased social interaction and increased repetitive grooming [87]. Spatial transcriptomics has revealed that the amyloid plaque niche serves as a hub for glial crosstalk: microglial activation drives recruitment and reaction of astrocytes and oligodendrocytes within a 10–40 µm zone around plaques. Receptor-ligand interaction analysis identifies strengthened signaling between microglia and astrocytes near plaques, including microglial Csf1 to astrocytic Csf1r, and astrocytic Apoe/Clu to microglial Trem2/Tyrobp. Plaque-associated microglia exhibit upregulated expression of complement components (e.g., C1qa), which is spatially correlated with synaptic loss and neuronal distress [42].

Metabolic and Oxidative Stress Interactions. Astrocyte-derived LCN2 is upregulated in response to Aβ oligomers, which in turn activates microglia, promotes iron accumulation, and induces oxidative stress in the hippocampus via pro-inflammatory cytokines (TNF-α, IL-6) and MMP-9-mediated blood–brain barrier disruption [88]. Inflammatory glial activation induced by systemic LPS leads to a significant increase in lipid peroxidation (TBARS) in the spinal cord; anti-inflammatory treatment with IL-1ra concurrently reduces both this oxidative stress marker and behavioral hypersensitivity [83]. Furthermore, glial pathology disrupts neuron–glial communication: knockdown of the glial-enriched protein DmMANF in Drosophila epithelial glia causes glial degeneration, a 30% decrease in capitate projections, and disorganized Na^+^/K^+^-ATPase distribution [89].

In summary, glial cells interact through cytokine, complement, and metabolic pathways to shape neural circuit function in BPSD, as comprehensively outlined in Table 1. Future investigations are warranted to examine how tripartite microglia-astrocyte-oligodendrocyte dynamics evolve across AD stages and how these multicellular networks contribute to specific BPSD clusters, potentially revealing novel intervention points targeting glial networks rather than individual cell types.

## 4. Glial Regulation of Neurotransmitter Systems in BPSD

Neurotransmitter system imbalance represents a core neurobiological basis of BPSD in AD, and glial cells, as key regulators of brain homeostasis, play a critical role in modulating the function of various neurotransmitter systems, thereby participating in the occurrence, progression, and potential intervention of BPSD.

### 4.1. Neurotransmitter Systems Imbalances in BPSD

Imbalances in multiple neurotransmitter systems constitute a key neurobiological substrate underlying BPSD in AD. A central and well-established hypothesis implicates cholinergic deficiency in the pathogenesis of specific BPSD clusters, such as psychosis, agitation, and apathy [90]. Pharmacological evidence supports this view: ChEIs such as donepezil have demonstrated modest yet significant benefits in ameliorating certain BPSD, particularly apathy, anxiety, and depression [90]. Conversely, exposure to medications with anticholinergic properties is associated with more frequent and severe BPSD, underscoring the delicate balance of this system [91].

Beyond the cholinergic system, monoamines imbalance are also critically involved. As comprehensively reviewed by Lanari et al. (2006), deficits in acetylcholine, dopamine, noradrenaline, and serotonin—together with dysfunction in brain regions such as the parahippocampal gyrus, dorsal raphe, and locus coeruleus—contribute significantly to the emergence of BPSD [92]. Specifically, noradrenergic hyperactivity has been linked to aggression, whereas dopaminergic deficits correlate with apathy [92,93]. Serotonergic dysfunction has been associated with symptoms like depression, anxiety, agitation, and aggression.

The inhibitory GABA system also exhibits marked disruption in AD. A systematic review and meta-analysis revealed that AD patients present lower GABA levels in the brain and cerebrospinal fluid, along with reductions in GAD65/67, GABA_A receptor, and GABA transporters [94,95]. Furthermore, glutamatergic-NMDA receptor dysfunction is implicated in both cognitive and behavioral symptoms of AD, with the NMDA receptor antagonist memantine showing some efficacy in managing BPSD [96].

Beyond these classical transmitters, purinergic signaling also plays a role: the P2X7 receptor is highly expressed on reactive microglia around Aβ plaques, and its activation drives IL-1β secretion and exacerbates neuroinflammation, representing an additional transmitter imbalance linked to behavioral symptoms [97,98].

Emerging multimodal neuroimaging evidence indicates that neurophysiological alterations in AD are topographically aligned with specific neurotransmitter systems, thereby providing a potential link to behavioral symptoms. Wiesman et al. (2024) combined magnetoencephalography with positron emission tomography to demonstrate that increases in delta/theta rhythms in cortical regions rich in dopaminergic and serotonergic receptors correlate with worse behavioral scores (e.g., delta–neurochemical alignment vs. behavior: t = −2.99, pFDR = 0.020) [99]. Given that glial cells dynamically regulate synaptic monoamine availability and receptor expression, these findings suggest that glia-driven neurotransmitter imbalances may underlie the neurophysiological signatures of BPSD.

### 4.2. Cholinergic System

Glial cells, particularly microglia and astrocytes, serve as crucial regulators of cholinergic homeostasis, and their dysregulation contributes substantially to BPSD in AD. Microglial α7nAChRs are upregulated in early AD pathology; their activation enhances Aβ phagocytosis and suppresses pro-inflammatory cytokines (TNF-α, IL-6), and promotes anti-inflammatory cytokines (IL-4, IL-10) [100,101]. In contrast, the purinergic receptor P2X7, which is highly expressed on reactive microglia surrounding Aβ plaques, exacerbates neuroinflammation upon activation [102]. The “cholinergic hypothesis of BPSD” posits that central cholinergic deficiency, resulting from degeneration of the nucleus basalis of Meynert, contributes to psychosis, apathy, and agitation [103]; this hypothesis is supported by the modest efficacy of ChEIs such as donepezil and galantamine in alleviating these symptoms [104,105]. Importantly, cholinergic projections activate glial α7nAChRs to suppress neuroinflammation via the Nrf2-HO1 antioxidant pathway [106,107]; AD-related cholinergic degeneration removes this protective brake, thereby exacerbating microglial inflammation [108]. This creates a vicious cycle whereby glial pro-inflammatory cytokines (IL-1β, TNF-α) further damage cholinergic neurons [109], amplifying both the cholinergic deficit and neuroinflammation.

Astrocytes support cholinergic function through choline transport and metabolism. The ASCOMALVA trial demonstrated that AD patients treated with donepezil (10 mg/day) combined with choline alphoscerate (1200 mg/day) for 24 months exhibited a significantly greater reduction in mood symptoms of BPSD compared wih those receiving donepezil alone [110]. Glial α7nAChRs are central to the “cholinergic anti-inflammatory pathway”, which inhibits pro-inflammatory cytokine synthesis [111]. This receptor binds with high affinity to antagonists such as α-bungarotoxin and with low affinity to acetylcholine [112]. Dysregulation of glial α7nAChR signaling thus links cholinergic dysfunction to glia-driven inflammation, with implications for depression and apathy [113]. A key regulation layer involves microRNA-mediated control of α7nAChR expression. Specifically, miR-98-5p, which is upregulated in AD patients and APP/PS1 mice, directly binds to the 3′UTR of Chrna7 mRNA to suppress α7nAChR protein translation. Notably, knockdown of miR-98-5p increased α7nAChR expression, activated the Ca^2+^/CaMKII pathway, restored synaptic proteins (PSD95, Synapsin-1), attenuated neuroinflammation through NF-κB inhibition, and enhanced the expression of Nrf2-targeted antioxidant genes (*HO-1*, *NQO-1*) [114]. Thus, glial cholinergic dysfunction acts as an active driver of neuroinflammation and BPSD, subject to epigenetic regulation.

Targeting glial cholinergic signaling offers therapeutic strategies. α7nAChR agonists, such as DMXBA, have demonstrated beneficial effects in preclinical models [100,115]. The efficacy of choline alfoscerate as an add-on therapy supports approaches aimed at enhancing astrocytic choline metabolism [110]. Anti-miR-98-5p oligonucleotides could restore α7nAChR expression, simultaneously enhancing synaptic function and reducing neuroinflammation [114]. Collectively, restoration of the glial–cholinergic interface—through α7nAChR agonists, enhancement of choline metabolism, or miRNA-based therapies—may yield disease-modifying treatments for BPSD.

### 4.3. Monoaminergic Systems

The 5-HT system modulates hippocampal excitability and neurogenesis via receptor subtypes expressed on both neurons and glia [116]. Glial cells exert a profound influence on BPSD by modulating serotonergic pathways, the dysfunction of which is linked to affective symptoms, agitation, and apathy in AD [117]. Microglia regulate serotonergic tone via multiple mechanisms. Specifically, Aβ oligomers induce depressive-like behavior through TLR4-dependent microglial activation, leading to increased TNF-α and reduced brain 5-HT levels; conversely, serotonin acts as a negative regulator of microglial activation [82]. Additionally, microglia-derived IL-1β and TNF-α modulate SERT activity: acute cytokine exposure enhances SERT function via p38-MAPK, whereas chronic inflammation reduces SERT availability, both of which contribute to serotonergic dysfunction underlying depression and apathy [47]. Moreover, activation of the kynurenine pathway via IDO depletes tryptophan and further compromises 5-HT synthesis, which represents an additional glia-involved mechanism driving depressive and apathetic symptoms in AD [118].

Astrocytes also contribute to monoaminergic dysregulation by expressing multiple 5-HT receptor subtypes. In the TgF344-AD rat model, a significant reduction in 5-HT_2A_ receptor density on striatal astrocytes was observed in aged AD rats. This reduction was accompanied by impaired 5-HT_2A_-dopamine system connectivity and reduced striatal dopamine release in young AD rats, even prior to overt cognitive decline [119]. This glia-mediated dysregulation of serotonergic-dopaminergic crosstalk represents a core mechanism underlying monoaminergic imbalance linked to BPSD in early AD. Astrocytes also express functional 5-HT_2B_ receptors, whose activation induces intracellular Ca^2+^ release via phospholipase C [120], and 5-HT_5A_ receptors that inhibit cAMP accumulation [121]. Furthermore, beyond canonical Gs-cAMP coupling, 5-HT_4_ receptors stimulate α-secretase activity via a Src-dependent PLC pathway, thereby promoting non-amyloidogenic sAPPα release and reducing Aβ secretion [122,123]. Additionally, astrocytic DRD2 signaling suppresses neuroinflammation through CRYAB; loss of this pathway exacerbates pro-inflammatory mediator release (e.g., IL-1β, IL-6) and correlates with nigral dopaminergic neuron degeneration in PD models, highlighting a direct glia-mediated link between monoamine disturbance and inflammatory-driven neuronal impairment [118]. Conversely, antagonism of the 5-HT_6_ receptor reduces Aβ formation partly by inactivating astrocytes and microglia [124], highlighting a reciprocal relationship in which glial 5-HT receptors both respond to and modulate AD pathology.

Thus, glial cells—through diverse 5-HT receptors (2A, 2B, 4, 5A, 6) and crosstalk with microglial cytokine-SERT pathways—orchestrate monoaminergic tone and influence amyloid metabolism, thereby forming a complex glial-serotonergic network underlying BPSD. Beyond classical amyloid and tau pathology, post mortem studies reveal significant reductions in nucleolar volume and total RNA within serotonergic and noradrenergic neurons in the brainstem of AD patients, which may be exacerbated by glia-mediated neuroinflammation [125,126,127]. Furthermore, specific BPSDs correlate with monoaminergic deficits that coincide with glial activation profiles: agitation/aggression in AD is associated with decreased serotonergic markers and increased striatal D2/D3 receptor availability [128,129], while apathy correlates with lower dopamine transporter binding in the frontal cortex [128,130]. These observations suggest that glial dysfunction may disrupt monoaminergic signaling, thereby shaping the presentation of BPSD. Targeting specific glial 5-HT receptors offers novel therapeutic opportunities beyond conventional serotonergic drugs. Astrocytic 5-HT_4_ receptors, which promote sAPPα release [122,123], suggest that 5-HT_4_ agonists could simultaneously alleviate affective symptoms and modify amyloid pathology. 5-HT_6_ antagonists under investigation for cognitive symptoms may exert benefits, at least in part, through glial inactivation [124]. Modulating microglial TLR4 signaling to reduce TNF-α-mediated serotonergic deficits [82] or targeting the p38-MAPK pathway to normalize SERT function [47] represent additional glia-centered strategies.

### 4.4. Glutamate/GABA Balance and Excitability

Astrocytes play a pivotal role in regulating glutamatergic and GABAergic transmission, and their dysfunction is closely associated with mood and behavioral disturbances in BPSD [131]. They maintain glutamate homeostasis primarily through GLT-1-mediated synaptic glutamate clearance and the glutamate–glutamine cycle, which is essential for balancing excitatory and inhibitory neurotransmission. Post mortem studies in AD patients have demonstrated reduced glutamate reuptake (SMD = −0.75) and impaired NMDA receptor function, accompanied by a selective decrease in the GluN2B subunit (SMD = −1.07) [132], findings indicative of marked astrocytic dysfunction in glutamate homeostasis. In 5xFAD mice, astrocytic synthesis of glutamine—an essential precursor for both glutamate and GABA—is significantly reduced, thereby impairing neuronal GABA production and destabilizing the synaptic excitatory/inhibitory (E/I) balance [133] and potentially underpinning neuronal hyperexcitability and BPSD-related symptoms such as agitation, anxiety, and sleep disturbances [134,135]. Notably, 5xFAD mice also exhibit biphasic changes in mGluR5: levels are decreased at 5 and 9 months but increased at 7 months, a pattern that coincides with periods of hyperactivity, and links glial-associated mGluR5 dynamics to BPSD [136]. Additionally, Aβ pathology disrupts this balance by downregulating astrocytic GLT-1, leading to elevated extracellular glutamate levels and hippocampal neuronal hyperexcitability [137]. Post mortem studies in AD further reveal reduced cortical GABA levels in the absence of altered glutamate concentrations, resulting in an elevated glutamate/GABA ratio that correlates with depressive symptoms, along with increased densities of GAT3/benzodiazepine receptor in the temporal cortex, which are linked to more severe depression [138]. Preclinical studies in CIH mouse models also show sex- and age-dependent changes in GABAergic neuron populations, underscoring the contribution of astrocytic failure to counterbalance neuronal hyperexcitability in BPSD [139]. Thus, converging evidence underscores that astrocytic disruption of glutamate/GABA homeostasis is a core pathogenic hub driving E/I imbalance and behavioral symptoms in BPSD.

Given this critical role, targeting astrocyte-mediated glutamate/GABA homeostasis offers promising therapeutic strategies for BPSD. The NMDA receptor antagonist memantine can normalize hippocampal glutamate levels and reduce anxiety- and depression-like behaviors in mouse models of AD [140]. Panek et al. (2024) developed a dual BuChE/GABA transporter inhibitor that specifically blocks astrocytic GAT3, effectively alleviating anxiety- and depression-like behaviors in mice [3]. Moreover, medium-chain fatty acids enhance astrocytic ketogenesis, and the resulting ketone body, β-hydroxybutyrate, may act as an extrasynaptic NMDA receptor inhibitor to counteract excitotoxicity [141,142,143]. These diverse pharmacological approaches—from receptor modulation to transporter blockade and metabolic intervention—collectively highlight the translational potential of restoring astrocytic E/I balance in BPSD.

Collectively, glial dysfunction disrupts cholinergic, monoaminergic, and glutamate/GABA signaling cascades, and consequent neurotransmitter dyshomeostasis acts as a critical intermediate driving the spectrum of BPSD manifestations in AD. The detailed relationships between neurotransmitter dyshomeostasis and glial regulation in BPSD are outlined in Table 2.

## 5. Glial Cells in Different BPSDs

**Figure 4:**
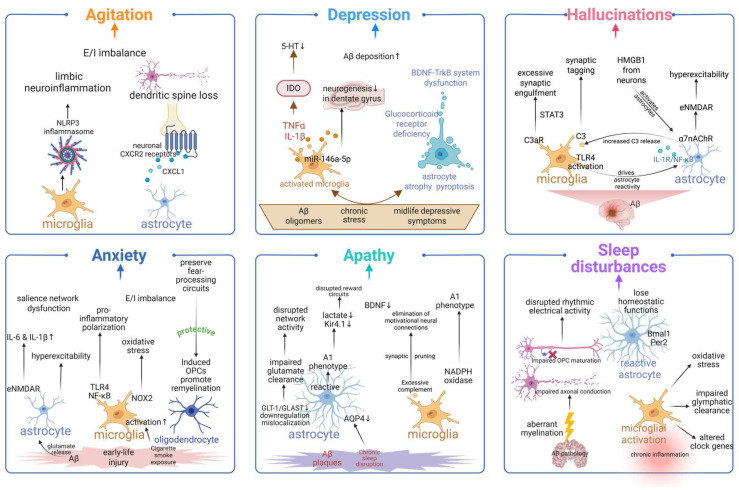
Roles of glial cells in different BPSDs. Arrows indicate causal or functional relationships: → represents a directional effect or pathway; ↑ denotes upregulation or increased activity; ↓ denotes downregulation or decreased activity. Each panel illustrates glial-mediated mechanisms underlying a specific BPSD. Created in BioRender. Hawthorn, M. (2026) https://BioRender.com/d2a4v8b (accessed on 17 May 2026).

BPSD in AD exhibits distinct clinical phenotypes, and glial cells play heterogeneous yet critical roles in the pathogenesis of various BPSDs, thereby providing a core cellular basis for symptom-specific pathophysiological mechanisms, as illustrated in Figure 4.

### 5.1. Agitation/Aggression

Agitation and aggression are among the most prevalent BPSDs and constitute a major driver of nursing home placement (OR = 1.10 per 10% increase) [144]. Convergent evidence implicates glial dysfunction as a key mechanism. Neuropathological studies indicate that agitation correlates more strongly with tau pathology than with amyloid burden, and lesion topography primarily involves limbic regions, including the amygdala and orbitofrontal cortex [17]. NLRP3 inflammasome-driven inflammatory responses contribute to aggressive behavior in animal models [145]. Additionally, reduced CB1 receptor function in AD, together with increased aggression observed in CB1 knockout mice, implicates the endocannabinoid system; given that CB2 receptors are primarily expressed on glia and modulate neuroinflammation, a glia-related mechanism is supported [146]. Reactive astrocytes exposed to Aβ release CXCL1, which in turn activates neuronal CXCR2 receptors, and induces dendritic spine loss and synaptic damage that persists even after Aβ removal [147]. Impaired glial clearance of pathological debris perpetuates neuroinflammation [148], and excitatory/inhibitory imbalance within limbic circuits destabilizes emotional control [149]. Human PET studies provide direct evidence: microglial activation is significantly associated with irritability severity, with agitation contributing 14.1% to the overall association between microglial activation and neuropsychiatric scores [35].

Current pharmacological interventions are associated with serious limitations; notably, off-label antipsychotics carry FDA black-box warnings [150]. Emerging glia-targeted strategies include NLRP3 inflammasome inhibitors, CB2 receptor agonists, blockade of the CXCL1-CXCR2 axis, and restoration of E/I balance via glial glutamate/GABA modulators [147,149]. The association observed in PET studies suggests that neuroinflammation imaging could guide anti-inflammatory interventions [35]. Shifting the therapeutic focus from symptomatic suppression toward glial-targeted disease modification may ultimately yield safer treatments for agitation and aggression in BPSD.

### 5.2. Depressive-like Symptoms

Depression is highly prevalent in AD (56% of cases). Although it is not a strong predictor of nursing home placement (OR = 1.03) [144], its co-occurrence with other psychiatric conditions is associated with more aggressive disease progression (HR = 2.81) [30]. Glial-mediated neuroinflammation is a core driver. Soluble Aβ oligomers induce depressive-like behavior in mice through microglial activation, the release of TNF-α and IL-1β, and the IDO pathway, which shunts tryptophan away from serotonin synthesis [151]. Chronic stress upregulates hippocampal TRPV1, which, in turn, activates microglial JAK2/STAT3 signaling; accordingly, TRPV1 inhibition ameliorates depressive behaviors [152]. Conversely, CX3CR1 deficiency promotes a protective microglial M2 phenotype and confers resilience to stress [44]. Astrocytes also play central roles: chronic stress induces astrocytic atrophy and pyroptosis [153]; astrocytic glucocorticoid receptor deficiency is sufficient to induce depressive phenotypes [154,155]; and dysfunction of the astrocyte-mediated BDNF-TrkB system represents a shared feature of depression and AD [156,157]. Genetic deletion of 5-HT_2B_ receptors produces an antidepressant-like phenotype [158,159]. Microglial depletion weakens astrocytic connexins [160], while astrocyte-derived IL-3 reprograms microglia to enhance Aβ clearance [161]. A specific mechanism involves microglial exosomal miR-146a-5p, which targets Klf4 mRNA in dentate gyrus neurons, thereby suppressing adult neurogenesis and inducing anhedonia [162]. Stress promotes Aβ deposition via microglial glycolysis and Kv1.3 channels [163], and peripheral CD8^+^ T cell expansion further engages glial inflammation [164]. Notably, midlife depressive symptoms—particularly loss of confidence, reduced warmth, and concentration difficulties—fully account for the depression-dementia association in individuals under 60 years of age [29].

Targeting these glial pathways offers novel therapeutic opportunities. Preclinical interventions include kaempferol, which restores AMPA receptor phosphorylation [153]; prebiotic supplementation targeting the IRS/PI3K/AKT pathway [165]; and restoring AIBP expression [166]. Furthermore, rTMS has been found to suppress neurotoxic astrocyte markers (C3, H2-T23), and serum GFAP correlates with depressive symptoms in MS patients [167]. Exosome-based or miRNA-targeted strategies [162] and blockade of the lactate/Kv1.3 pathway [163] could break the depression → Aβ cycle. Early, glial-targeted preventive strategies are warranted given that midlife depressive symptom clusters predict dementia risk [29] and psychiatric multimorbidity dramatically increases the hazard [30]. Restoring glial homeostatic functions—by modulating microglial metabolic reprogramming, astrocytic neurotrophic support, and exosomal communication—may alleviate depressive symptoms and modify AD progression.

### 5.3. Hallucinations

Visual hallucinations in AD are strongly associated with LRP that co-occurs with Alzheimer’s pathology [17]. Within this context, glial dysfunction disrupts sensory integration and circuit stability. Microglial activation leads to increased complement C3, which tags synapses for phagocytic engulfment—a process that may manifest as perceptual disturbances [168]. Dysfunctional microglial pruning in other contexts has been shown to produce aberrant synaptic connectivity and altered E/I balance, suggesting that similar mechanisms may operate in AD [169]. Astrocytic α7nAChRs are overexpressed in AD due to Aβ pathology, leading to abnormal glutamate release and extrasynaptic NMDA receptor hyperactivation, resulting in network hyperexcitability [170]. Cortical spreading depolarization studies reveal that neuronal hyperactivity initiates a cell-specific inflammatory cascade: neurons release HMGB1, activating astrocytes to drive a transient pro-inflammatory response while microglia adopt a repair-oriented profile; chronic pathology may corrupt this glial programming, disrupting cortical inhibition [171]. A key pathway involves astrocyte-microglia crosstalk via the IL-1R/C3/C3aR axis: chronic stress activates astrocytic IL-1R/NF-κB signaling, increasing C3 release and triggering microglial STAT3-dependent excessive synaptic engulfment [172]. TLR4-mediated activation of microglia and monocytes also drives astrocyte reactivity, impairing synaptic density and network excitability [173].

Therapeutic strategies targeting glial modulation have shown promise. Notably, strategies aimed at synaptic regeneration, without altering core proteinopathy, can restore function, suggesting that glia-supported synaptic repair may occur independently of amyloid/tau clearance [174]. Spermidine modulates DAM by enhancing phagocytosis and suppressing inflammasome activation, thereby reducing neuroinflammation and hyperexcitability [175]. Targeted overexpression of mitochondrial UCP4 in hippocampal astrocytes prevents neuronal hyperexcitability and preserves dendritic architecture [176]. Modulating microglial complement signaling, normalizing astrocytic α7nAChR activity, or enhancing astrocytic mitochondrial bioenergetics may help stabilize sensory circuits. A shift from symptomatic antipsychotic treatment toward symptomatic antipsychotics toward glial-targeted strategies offers a novel approach for managing hallucinations in AD- related BPSD.

### 5.4. Anxiety

Anxiety in AD is increasingly linked to glial modulation within fear and emotional salience circuits. A TSPO-PET study demonstrated that microglial activation correlated with overall neuropsychiatric symptoms, with regional involvement of the anterior cingulate cortex—a salience network node—suggesting that glia-driven neuroinflammation creates a vulnerable substrate for anxiety [35]. Aβ-driven astrocytic glutamate release activates eNMDARs, thereby elevating IL-6 and IL-1β and forming an excitotoxic-inflammatory axis implicated in anxiety-like behaviors [141,177]. In a neonatal hypoxic–ischemic model that progresses to AD-like pathology, early injury induced persistent anxiety and dysregulation of inflammatory genes shared with AD [33]. Moreover, cigarette smoke exposure in a COPD model induced anxiety, accompanied by increased microglial activation (enlarged cell area) and upregulation of pro-inflammatory genes (*Il6*, *Il1β*) in amygdala and hippocampus [178]. Conversely, overexpression of thymosin β4 in APP/PS1 mice reversed the pro-inflammatory polarization of microglia and astrocytes, downregulated TLR4/NF-κB signaling, and alleviated anxiety-like behaviors [179]. Hyperactive enteric glia exhibit intrinsic hyperexcitability via PGE_2_ and connexin-43 hemichannels [180]; by analogy, reactive glia in limbic circuits may similarly lower the threshold for fear responses. Furthermore, induced OPCs promoting new myelin formation with clemastine improved remote fear memory recall in mice, suggesting that enhanced oligodendrogenesis can support fear-processing circuits [181]. Collectively, these findings indicate that anxiety in AD arises from convergent glial mechanisms—microglial inflammation (TSPO, TLR4/NF-κB), astrocyte-mediated eNMDAR hyperactivation, glial hyperexcitability via connexin hemichannels, and oligodendrocyte dysfunction—that destabilize limbic and salience networks.

Targeting these glial pathways offers promising therapeutic strategies. The ketone body βHB, whose production is enhanced by astrocyte metabolism, functions as an eNMDAR inhibitor; medium-chain fatty acids (e.g., coconut oil) have been associated with reduced anxiety in AD [143,182,183]. Chemogenetic activation of hippocampal astrocytes expressing hM3D(Gq) in 5xFAD mice restored decreased anxiety levels and reduced amyloid plaque burden [184]. The NOX2 inhibitor apocynin alleviated anxiety and restored microglial morphology [178]. Thymosin β4 overexpression [179] and strategies that promote oligodendrocyte progenitor cell-mediated remyelination [181] further expand the therapeutic landscape. Normalizing astrocytic glutamate/eNMDAR signaling, inhibiting microglial TLR4/NF-κB or NOX2 pathways, and preserving oligodendrocyte-supported circuit integrity may address the root mechanisms of anxiety in AD-related BPSD.

### 5.5. Apathy

Apathy, characterized by diminished motivation, is increasingly recognized as a consequence of reduced glial support for neuronal activity. Astrocytes maintain synaptic homeostasis via GLT-1/GLAST-mediated glutamate clearance; in AD, reduced expression and mislocalization of these transporters, particularly in the vicinity of Aβ plaques, disrupt network activity and may underlie diminished motivation [7]. Reactive astrogliosis and the shift toward a neuroinflammatory A1 phenotype impair metabolic support (e.g., lactate) and ion regulation (Kir4.1, α2-NKA), thereby disrupting reward-processing circuits. Concurrently, microglial dysregulation and excessive complement-mediated synaptic pruning may eliminate motivational neural connections [185]. Glia-driven oxidative stress—mediated by microglial NADPH oxidase and compromised astrocytic antioxidant responses, further damages synaptic membranes [186]. A CSF biomarker study identified distinct astrocyte clusters based on GFAP, YKL-40, and AQP4 levels; post hoc analysis revealed that apathy/indifference differed across these clusters, with a low GFAP/AQP4 profile—reflecting relatively preserved glial capacity—correlating with milder apathy [57]. Chronic sleep disruption reduces astrocytic glutamate uptake and BDNF release, thereby disrupting plasticity [187]. Pain and unmet physiological needs, both associated with apathy, may trigger microglial activation and astrocytic metabolic failure [188].

Therapeutic strategies targeting glial and cholinergic functions are emerging. The ASCOMALVA trial demonstrated that donepezil combined with choline alfoscerate reduced apathy scores on the NPI [110]. In 5xFAD mice, apathy-like behaviors (e.g., nest building and burrowing) emerge at 6 months of age, precede memory deficits, and correlate with soluble Aβ42 levels and plaque burden in the prefrontal cortex and hippocampus [189]. Future strategies include upregulation of GLT-1, lactate supplementation, inhibition of complement-mediated pruning, and targeting AQP4 to enhance glymphatic function. Restoring glial homeostatic functions—namely glutamate clearance, metabolic support, and synaptic integrity—holds promise for addressing apathy in AD-related BPSD.

### 5.6. Sleep Disturbances

Sleep disturbances in AD may arise from disrupted glial regulation of circadian rhythms and network integrity. Subcellular proteomics reveals paranodal pathology and aberrant myelination without overt myelin loss, thereby impairing axonal conduction and network synchronization [190]. Oligodendrocyte progenitor cells and healthy oligodendrocytes sustain the rhythmic electrical activity of sleep–wake circuits; impaired OPC maturation disrupts neural timing [180]. Astrocytes and microglia express intrinsic circadian clocks and modulate sleep–wake cycles via neuroinflammatory signaling, metabolic coupling, and synaptic plasticity. In chronic insomnia, glial activation promotes oxidative stress, impairs glymphatic clearance, and alters clock gene expression (e.g., *Bmal1*, *Per2*) [187]. Reactive glia in circadian centers may similarly lose their homeostatic functions. In Drosophila, glial-specific downregulation of DmMANF (but not neuronal knockdown) altered sleep/activity patterns (reduced daytime activity and increased nighttime activity) and was accompanied by ultrastructural degeneration of epithelial glia [89]. Thymosin β4 overexpression in APP/PS1 mice reversed the pro-inflammatory polarization of microglia and astrocytes via NF-κB inhibition, thereby restoring glial homeostasis, which is critical for circadian integrity [179]. In IDH-mutant astrocytoma, reactive glia expressing SPP1, IL-1β, and CD44 create an immunorestrictive microenvironment [191], illustrating how glia-driven neuroinflammation could disrupt suprachiasmatic nucleus function. Chemogenetic activation of astrocytes rescued broader neurological function [184], and optogenetic stimulation of astrocytes restored slow brain rhythms and reduced amyloid pathology [192].

Future strategies include promoting oligodendrocyte differentiation and remyelination (e.g., clemastine), normalizing glial circadian clocks via clock gene modulators, and enhancing glymphatic function. Restoring glial homeostasis—encompassing myelin integrity, astrocytic metabolic support, microglial inflammatory balance, and clock gene regulation—holds promise for addressing sleep disturbances in AD-related BPSD.

## 6. Molecular Mechanisms of Glial Cell Involvement in BPSD

**Figure 5:**
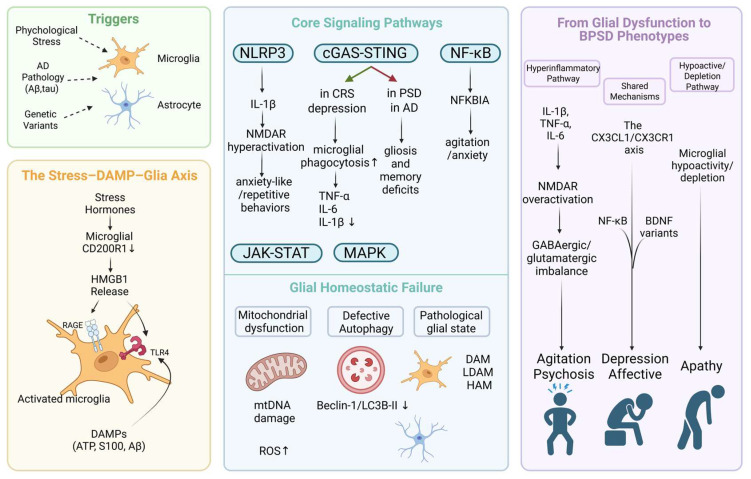
Molecular mechanisms of glial cell mediation in BPSD. Color coding: green (Triggers), orange (Stress-DAMP-Glia Axis), light blue (Signaling/Homeostasis), purple (BPSD pathways). Arrows: → denotes a causal pathway; ↑/↓ denotes upregulation/downregulation. Created in BioRender. Hawthorn, M. (2026) https://BioRender.com/d2a4v8b (accessed on 17 May 2026).

Beyond classic neuroinflammatory pathways, glial involvement in BPSD also encompasses genetic variants that modulate cellular stress and one-carbon metabolism. The MTHFR 677T variant, which reduces enzyme activity and increases homocysteine levels, has been linked to delusions in AD patients [31]; elevated homocysteine induces oxidative stress and mitochondrial dysfunction in astrocytes and microglia, thereby contributing to neurotoxicity and specific BPSDs such as psychosis. Thus, glial molecular mechanisms—ranging from genetic variants to cellular stress responses—translate AD pathology into distinct behavioral symptoms. Understanding these pathways is critical for developing mechanism-based interventions. The following sections detail specific DAMPs, signaling cascades, cytokine networks, pathological cellular states, and genetic modulators driving glial dysfunction in BPSD, as summarized in Figure 5.

### 6.1. The Stress–DAMP–Glia Axis

In AD, psychological and physiological stressors induce sterile neuroinflammation via DAMPs. Stress hormones downregulate microglial CD200R1, leading to the release of the alarmin HMGB1, which signals through TLR4 and RAGE to induce a pro-inflammatory phenotype [193]. Other DAMPs (e.g., ATP, S100 proteins, aggregated Aβ) activate microglia mainly via TLR2 and TLR4, thereby driving neuronal network dysfunction through the production of reactive oxygen and nitrogen species [194,195]. Astrocyte-derived APOE binding to LRP8 on inhibitory neurons, as well as oligodendrocyte-derived APP engaging GPC1 on excitatory neurons, represent additional DAMP-receptor interactions involving AD risk genes [37]. Chronic stress amplifies this axis: in 5xFAD mice, social isolation combined with unpredictable stress increased Aβ plaque load and pushed glial responses toward dysfunctional states [196]. Thus, the DAMP–glia axis constitutes a key molecular interface linking psychological stress and AD pathology to glia-driven BPSD. Targeting DAMP receptors (e.g., TLR4 antagonists, HMGB1-neutralizing antibodies) holds therapeutic promise. A critical caveat is that most DAMP studies have been derived from AD models; whether specific DAMP signatures correlate with distinct BPSD clusters (e.g., agitation vs. psychosis) remains unknown. Future research should map DAMP profiles to behavioral phenotypes, potentially enabling biomarker-driven therapeutic strategies, such as TLR4 antagonists or HMGB1-neutralizing antibodies. Collectively, understanding the DAMP–glia axis may reveal novel targets for interrupting the stress–inflammation–behavior loop in BPSD.

### 6.2. Core Signaling Pathways

Key intracellular signaling pathways in glia drive BPSD-related neuroinflammation by translating extracellular stimuli into functional responses. The cGAS-STING pathway acts in a context-dependent manner: in chronic restraint stress-induced depression, STING activation (via 2′3′-cGAMP) enhanced microglial phagocytosis, reduced TNF-α, IL-6, and IL-1β levels, and improved behavior [197]; by contrast, in post-stroke depression, its overactivation proved detrimental, an effect reversed by hydrogen sulfide [198]; in AD models, Aβ, tau and APOE ε4 activate this pathway in microglia, with STING inhibition alleviating gliosis and memory deficits [199]. The NLRP3 inflammasome is also pivotal. A 2025 study demonstrated microglial NLRP3 activation induces anxiety-like and repetitive behaviors via IL-1β-mediated NMDAR hyperactivation, an effect normalized by NMDAR or IL-1 receptor blockade [200]; notably, 40% patients with systemic inflammation exhibit residual neuropsychiatric symptoms [201]. The JAK-STAT pathway mediates DAMP and cytokine signals: CSDS-induced depression increased hippocampal JAK2/STAT3 phosphorylation, which was linked to microglial activation/IL-6/IL-1β levels, and this effect was reversed by TRPV1 inhibition [152]. This pathway also regulates astrocytic scarring and serotonin receptors [194,202]. NF-κB is implicated in affective BPSD: NFKBIA was identified as a cell stress hub gene [5], and AD snRNA-seq revealed elevated NF-κB activity in prefrontal inhibitory neurons (linked to agitation/anxiety) [37]. MAPK (p38/JNK activated in Aβ-exposed astrocytes [203]) and lactate-Kv1.3 (microglial lactate activates Kv1.3, promoting Aβ exosome release [163]) also contribute, with AKT/MAPK dysregulation linked to glial inflammation and behavioral deficits [33]. These interconnected pathways form a glial signaling network underlying BPSD; their druggability (e.g., approved JAK inhibitors, preclinical NLRP3/STING modulators) highlights therapeutic potential, though cGAS-STING’s context-dependence necessitates precise modulation over simple inhibition to optimize efficacy.

### 6.3. Neuron–Glia and Peripheral-Central Cytokine Networks

The CX3CL1/CX3CR1 axis mediates direct neuron-microglia communication in BPSD. In a chronic unpredictable stress model, genetic ablation of microglial CX3CR1 reduced hippocampal/peripheral levels of IL-1β, IL-6, and TNF-α, promoted M2 microglial polarization, preserved synaptic function, and alleviated depressive-like/cognitive deficits [44]. Activated glia release IL-1β, TNF-α, and IL-6, which impair dopaminergic/serotonergic signaling and the glutamatergic/GABAergic balance, thereby contributing to BPSD-related symptoms [204]. The observation that microglial reduction is specific to apathy suggests subtype-specific glial involvement. Specifically, pro-inflammatory cytokines may drive hyperactive BPSDs (e.g., agitation) via NMDAR modulation, whereas microglial hypoactivity/depletion may underlie apathy—a key distinction for precision therapy. Cytokine networks also link peripheral immunity to central glial activation: in stress-susceptible 5xFAD mice, CyTOF detected increased numbers of CD8^+^/CD4^+^ memory T cells, along with hippocampal upregulation of Th17/antigen-presentation pathways and reduced ZO-1 expression, indicating blood–brain barrier disruption [164]. Therapeutic opportunities include CX3CR1 agonists, IL-1/TNF-α inhibitors [200,204], and the currently understudied field of blood–brain barrier repair. Longitudinal cytokine profiling in patients is critical, as combinatorial cytokine signaling determines BPSD phenotypes, and multi-omics integration is needed to develop symptom-specific anti-inflammatory therapies.

### 6.4. Pathological States and Homeostatic Failure in BPSD

Glial homeostatic failure underlies BPSD and is driven by intracellular stressors, including oxidative stress, mitochondrial dysfunction, defective autophagy, and dysregulation of organelle contact. In dementia patients, peripheral CAT activity correlated with baseline BPSD severity (BEHAVE-AD score, *p* = 0.024), and higher CAT levels predicted greater improvement in BPSD following treatment with sodium benzoate [205], aligning with astrocytic CAT dysfunction in AD-related oxidative stress [206,207]. Microglial mitochondrial dysfunction observed in AD involves mtDNA damage (a DAMP that activates the cGAS-STING/NLRP3 pathways [208]), induced by Aβ/p-Tau glycolytic shift, and defective mitophagy. Activation of α7nAChR in glia suppresses ROS via the Nrf2 pathway, whereas Aβ-induced ROS is inhibited by nicotinic stimulation [106,107,108,209,210]. Defective autophagy has also been linked to BPSD. SAMP8 mice exhibited autophagic deficits (reduced Beclin-1/LC3B-II levels) along with BPSD-like behaviors [211], whereas 5xFAD microglia had impaired autophagy-lysosomal function, a defect reversed by microglial TFEB overexpression [196]. In 3xTg-AD astrocytes, ER-mitochondria contact sites were shortened to approximately 8–10 nm, a phenotype rescued by amorolfine [199,212]. Furthermore, glial knockdown of DmMANF induced ER stress-driven degeneration [89]. In AD, microglia adopt pathological states (e.g., DAM, LDAM, HAM) that disrupt neural circuits [56,213,214]. These interconnected pathological states collectively compromise glial homeostasis; therapeutic strategies targeting glial health—such as Nrf2 activators, mitophagy inducers, TFEB enhancers—rather than focusing solely on inflammation, offer a more fundamental approach to treating BPSD, with state-specific targeting (e.g., DAM/LDAM) meriting further exploration.

### 6.5. Genetic Variants

Genetic variants regulating glial function predispose to specific BPSDs in AD: beyond TREM2/CD33 (glial phagocytosis/neuroinflammation), neurodevelopment-related gene SNPs correlate with BPSD. Porcelli et al. (2016) found BDNF (rs6265/rs11030104), ST8SIA2 (rs3759917), and C15orf32 (rs4777989) variants linked to peripheral inflammatory markers (IL-6, CRP, ICAM-1), with BDNF/ST8SIA2/NCAPG2 variants associated with depression and PLA2G4A/SP4/C15orf32/BDNF variants with psychosis [51]. The MTHFR 677T variant (elevating glial oxidative stress) was linked to delusions in AD patients [31]. Multi-omics show shared AD/mental illness dysregulation in microglia-related myeloid activation and synaptic function [215]. Collectively, these variants connect genetic susceptibility to glial dysfunction and distinct BPSDs, supporting a personalized medicine approach—MTHFR 677T carriers may benefit from folate/B12 supplementation, while BDNF/ST8SIA2 variants could guide glia-modulating therapy, with genetic biomarker panels enabling BPSD risk stratification.

## 7. Glia-Based Therapeutics for BPSD in AD

Conventional symptomatic treatments for BPSD have limited efficacy. For example, a trial of SSRIs in MCI patients failed to alter AD progression, cognitive decline, or AD biomarkers [216], whereas atypical antipsychotics have demonstrated only 28% symptom improvement and a 53% adverse event rate, accompanied by a black-box warning for increased mortality [217]. These therapeutic gaps suggest that BPSD is sustained by mechanisms beyond neuronal synaptic deficits, most likely persistent neuroinflammation and glial homeostatic dysfunction. In this context, high-risk symptom profiles (e.g., midlife social-affective blunting [29]) support precision approaches. Consequently, glia-targeted interventions are promising. Examples include Kampo medicine Yokukansan (which enhances astrocytic glutamate transporters, suppresses microglial activation, promoting oligodendrocyte differentiation [218,219,220,221,222]), cholinesterase inhibitors that depend on astrocytic choline uptake [110], nanotherapeutic R@AClipo (co-delivering TREM2 agonist and riluzole [137]), and clinical trials such as microglial inhibitor EI-1071 (NCT06745583). These examples highlight the translational potential of glia-based strategies for addressing both cognitive and behavioral deficits in AD [215]. Further glia-based therapeutic strategies are detailed in the following sections.

### 7.1. Microglial Anti-Inflammatory

Microglial pro-inflammatory pathways are key drivers of BPSD. Clinical biomarker studies have shown that microglial activation independently correlates with neuropsychiatric symptoms in AD, such as irritability and agitation, even after adjusting for amyloid and tau pathology [35]. Preclinically, CX3CL1-CX3CR1 axis modulation promotes microglial M2 polarization, reduces IL-1β/IL-6/TNF-α levels, and alleviates stress-induced depressive-like behaviors [44]; NLRP3 inflammasome activation in microglia induces IL-1β-mediated NMDAR hyperactivation and repetitive behaviors, an effect reversed by IL-1 receptor blockade or memantine [200]. By contrast, the STING inhibitor H-151 reduces microglial synaptic engulfment and gliosis in AD mice [199]. Microglial Kv1.3 upregulation under depressive conditions is a validated target; conditional knockout of Kv1.3 reversed stress-induced Aβ deposition and cognitive deficits [163]. Atypical antipsychotics (e.g., clozapine, quetiapine) exert part of their BPSD efficacy by inhibiting microglial cytokine release and the NLRP3/NF-κB pathways [223,224]. Beyond these mechanisms, targeting the purinergic P2X7 receptor on microglia offers another anti-inflammatory route. The selective brain-penetrant antagonist JNJ-54175446 blocks ATP-induced inflammasome activation and IL-1β release, attenuates anhedonia in preclinical models, and was safe and well-tolerated in a clinical proof-of-concept study in major depressive disorder, suggesting potential for treating BPSDs such as apathy and anhedonia [225]. Additionally, the anti-Alzheimer’s drug GV-971 (sodium oligomannate) remodels the gut microbiota to increase short-chain fatty acids (propionate, butyrate), which suppress pro-inflammatory microglial polarization via MAPK blockade, positioning it as a candidate for both cognitive and behavioral symptoms in AD [226]. Diverse anti-inflammatory strategies are currently under development, including NLRP3 inhibitors (MCC950, dapansutrile [227]), CSF1R inhibitor EI-1071 (Phase II, NCT06745583), nasal foralumab [228], Kv1.3 blockade, P2X7 antagonism, and microbiota-modulating agents. These approaches offer complementary options to amyloid-targeting therapies for the relief of behavioral symptoms in BPSD.

### 7.2. Astrocyte Homeostasis Restoration

Restoring astrocyte homeostatic functions—namely glutamate clearance, antioxidant defense, and metabolic support—represents a promising therapeutic direction for BPSD. In AD, reduced astrocytic glutamate reuptake (in the absence of changes in EAAT2 protein levels) leads to excitotoxicity and agitation [132]; astrocyte dysfunction also contributes to neuropsychiatric disorders such as schizophrenia, where antipsychotics (e.g., clozapine) modulate astrocyte glutamate clearance and synaptic remodeling [229]. Existing antidepressants (fluoxetine, amitriptyline) exert BPSD benefits partly by upregulating astrocytic release of GDNF/BDNF and by enhancing connexin 43-mediated gap junction communication [230,231,232]; meanwhile, ketamine modulates astrocyte-centric BDNF-TrkB signaling deficient in depression and AD [156,233]. Therapeutically, natural metabolites such as Urolithin A alleviate depression/anxiety-like behaviors via AMPK/CREB/BDNF signaling [234]; antipsychotics (risperidone) increase astrocytic glutamate uptake and glutathione levels, thereby reducing NF-κB-mediated inflammation [235,236,237,238]; and resveratrol adjuvants sustain astrocyte homeostasis during antipsychotic treatment [223]. Memantine normalizes hippocampal glutamate levels and reduces anxiety-like behaviors, whereas clozapine modulates astrocytic glutamate handling [223]; furthermore, chemogenetic activation of hippocampal astrocytes in 5xFAD mice rescues LTP, memory, and BPSD-like deficits while reducing Aβ plaque load [184]. Collectively, these strategies—including dietary metabolites, repurposed drugs, and functional enhancement—target astrocyte homeostasis to alleviate BPSDs.

### 7.3. Oligodendrocyte and Myelin Repair

Promoting oligodendrocyte differentiation and remyelination represents a promising approach for alleviating BPSDs, particularly affective symptoms such as apathy and social withdrawal. Preclinically, oral administration of clemastine (an antimuscarinic promoting oligodendrocyte differentiation) rescued social avoidance and prefrontal hypomyelination in a mouse model of social isolation-induced depressive-like behavior. This effect was mediated by an increase in mature oligodendrocytes and by epigenetic regulation of H3K9me3 [77]; additionally, BDNF protects oligodendrocyte precursor cells against Aβ_1–42_-induced cell death and promotes their proliferation/differentiation in vitro [239]. Therapeutically, Cognito Therapeutics’ Spectris™ device (40 Hz gamma light/sound stimulation) preserved white matter integrity and reduced emotional stability/social engagement decline (total scores −0.59 vs. −15.12 in the sham group, *p* = 0.0006) in an open-label extension study [239]. Repurposed drugs (antihistamines such as clemastine) and non-invasive neuromodulation, together with BDNF-based neurotrophic support, highlight the therapeutic potential of oligodendrocyte/myelin-targeting for BPSD.

### 7.4. Glial–Neuronal Crosstalk Modulation

Given the limitations of current BPSD treatment—antipsychotics carry a black-box warning for increased mortality in dementia and only modest efficacy [150]—targeting glial-neuronal crosstalk is urgently required. Memantine (an NMDA receptor antagonist) improved BPSD-like behaviors (anxiety, depression) in AD mice, correlating with astrocyte-mediated hippocampal glutamate regulation [140]. Aβ/tau activate astrocytic IDO1, thereby reducing glycolysis/lactate production via AhR; the brain-penetrant IDO1 inhibitor PF06840003 restored astrocyte metabolic support, hippocampal glucose metabolism, and behavioral deficits in AD models [240]. Therapeutically, LM11A-31 (a modulator of the p75 neurotrophin receptor) reduced synaptic degeneration, glial abnormalities, and BPSD-like deficits in mouse models, with consistent results from the Phase 2a clinical trial [241]. The nanotherapeutic R@AClipo, which co-delivers the TREM2 agonist COG1410 and riluzole, enhanced microglial Aβ clearance, reduced glutamate accumulation, and mitigated BPSDs (including agitation and sleep disturbances) in AD mice [137]. Collectively, these strategies targeting glial-neuronal crosstalk address core mechanisms of BPSD and cognitive deficits.

### 7.5. Gene and Cell Therapies

Emerging gene-editing and cell replacement technologies offer novel glia-targeted strategies for BPSD. Patient-derived iPSCs enable individualized modeling of BPSD; for instance, hindbrain organoids derived from AD patients exhibit patient-specific responses to SSRIs [242]. GRP transplantation into AD rat models reversed anxiety-like and depressive-like behaviors, stimulated endogenous neurogenesis/gliogenesis, and altered plasticity-related proteomes [243]. CRISPR/Cas9 editing corrected TREM2 loss-of-function variants in human iPSC-derived microglia, thereby restoring phagocytic capacity and reducing pro-inflammatory cytokine release [244]. Hematopoietic cell transplantation replaced Trem2-deficient microglia with wild-type myeloid cells, restoring Aβ clearance and mitigating BPSD-like deficits in AD mice [245,246]. These approaches—iPSC modeling, GRP transplantation, CRISPR editing, and hematopoietic replacement—leverage gene/cell therapies to correct glial dysfunction for BPSD relief.

### 7.6. Emerging Agents for BPSD

Psychedelic compounds—particularly psilocybin and 5-MeO-DMT—are emerging candidates for the treatment of BPSD, with glial mechanisms underpinning their potential therapeutic effects. Psilocybin metabolite psilocin suppresses microglial TNF-α production, enhances BDNF via 5-HT_2A_ and AhR signaling, and inhibits microglial ROS/NO production in a 5-HT_2_ receptor-dependent manner [247,248]; similarly, 5-MeO-DMT reduces astrogliosis in an Aβ-injected mouse model of AD [248]. Two BPSD-relevant trials have been conducted or are ongoing: NCT04123314 (ongoing) evaluates psilocybin (15/25 mg) for depression in patients with MCI/early AD, while NCT06812221 (completed Phase I/II) tested sublingual 5-MeO-DMT for anxiety/depression in mild-to-moderate AD. CBD represents another candidate with glial-modulating properties. It acts on CB2 receptors and non-cannabinoid targets (e.g., 5-HT_1A_, PPARγ) to exert anti-inflammatory and anxiolytic effects. Two ongoing trials are evaluating CBD for BPSD: NCT04075435 (open-label study of a high-CBD/low-THC sublingual solution for anxiety/agitation in MCI or mild-to-moderate AD) and NCT04436081 (randomized, placebo-controlled crossover trial of THC-free CBD oil for agitation in AD). Furthermore, a Phase 2b trial of GH001 (inhaled 5-MeO-DMT) in 81 patients with treatment-resistant depression demonstrated robust efficacy. The MADRS score reduction −15.5 vs. placebo (*p* < 0.001; Cohen’s d = −2.0), 57.5% remission at day 8, and a HAM-A reduction –10.0, no severe adverse events. Although focused on TRD, the 5-HT_1A_-mediated effects of GH001 suggest indirect glial modulation as astrocytes/microglia express 5-HT receptors, and 5-HT_1A_ activation reduces neuroinflammation. Overall, the mood effects and anti-inflammatory actions of psychedelics and CBD position them as novel candidates for BPSD, but further validation in AD models and well-controlled clinical trials is required.

The specific glia-based therapeutics for BPSD are summarized in Table 3.

## 8. Conclusions

Over the past decade, BPSD in AD has been redefined: from a consequence of neuronal loss to a pathology actively driven by glial dysfunction. Microglia, astrocytes, and oligodendrocytes orchestrate neuroinflammation, synaptic pruning, metabolic support, and neurotransmitter homeostasis. Their failure—through excessive inflammation, impaired glutamate clearance, cholinergic dysfunction, or defective myelination—directly contributes to specific BPSDs. This review has mapped the molecular pathways linking glial pathology to behavioral phenotypes. Restoring glial homeostasis represents a rational, disease-modifying therapeutic strategy. However, most current evidence links glial pathology to BPSD through association rather than causation, leaving it unclear whether specific glial states are sufficient and necessary drivers of distinct phenotypes or merely epiphenomena of neuronal degeneration. Interdisciplinary efforts integrating glial biology, behavioral neuroscience, and biomarker development are required to translate these insights into effective treatments. Over the next decade, advances in single-cell technologies, biomarker discovery, and glia-targeted therapeutics may enable more precise and mechanism-based interventions for specific BPSD domains in AD.

## Figures and Tables

**Table 1:** Pathological cell interactions underlying BPSD.

Cell Pairs	Routes	Relevance to BPSD
Microglia → Serotonergic neurons	AβO binding to microglial TLR4 → TNF-α release	Induces depressive-like behavior via decreased brain 5-HT [82].
Serotonergic neurons → Microglia	5-HT acts as a negative regulator	Prevents AβO-induced microglial activation and TNF-α elevation [82].
Microglia ↔ Astrocytes	IL-1β positive feedback loop (COX-2, PGE2)	Drives systemic inflammation-induced glial activation and behavioral hypersensitivity [83].
	IL-1ra	Blocks the IL-1β loop, reducing glial activation and ameliorating behavioral hypersensitivity [83].
	Release of IL-1α, TNF-α, C1q	Drives astrocytes toward a detrimental, neuroinflammatory A1 phenotype [84].
Astrocytes → Microglia	Release of GDNF	Modulates microglial activity [85].
Microglia → Neurons (ACC)	CR3 deficiency (impaired complement-mediated clearance)	Leads to increased neuronal density and enhanced local functional connectivity in adulthood [86].
Microglia → Neurons (prefrontal-hippocampal)	CX3CR1 deficiency (defective fractalkine signaling)	Causes reduced synaptic density, diminished functional connectivity, and BPSD-like behaviors (decreased social interaction, increased repetitive grooming) [87].
Microglia → Astrocytes (plaque niche)	Microglial Csf1 → astrocytic Csf1r	Strengthened signaling between microglia and astrocytes within 10–40 µm of amyloid plaques [42].
Astrocytes → Microglia (plaque niche)	Astrocytic Apoe/Clu → microglial Trem2/Tyrobp	Strengthened signaling between astrocytes and microglia near plaques [42].
Microglia → Neurons (plaque niche)	Upregulated complement component C1qa	Spatially correlated with synaptic loss and neuronal distress around plaques [42].
Astrocytes → Microglia	Astrocytic LCN2 (upregulated by Aβ oligomers) → activates microglia, promotes iron accumulation, induces TNF-α/IL-6	Causes hippocampal oxidative stress and MMP-9-mediated blood–brain barrier disruption [88].
Microglia/Astrocytes (spinal cord)	LPS-induced lipid peroxidation (TBARS); reversed by IL-1ra	Serves as an oxidative stress marker that correlates with behavioral hypersensitivity [83].
Glial cells (Drosophila epithelial glia) → Neurons	Knockdown of glial-enriched protein DmMANF	Leads to glial degeneration, 30% decrease in capitate projections, and disorganized Na^+^/K^+^-ATPase distribution, disrupting neuron–glia communication [89].

Notes: Arrows denote interaction direction: → indicates a unidirectional effect from the former to the latter cell type; ↔ indicates bidirectional communication between the two cell types. Arrows in the routes column represent sequential steps in signaling pathways.

**Table 2:** Neurotransmitter system imbalances and glial regulatory mechanisms in BPSD.

Neurotransmitter System	Glial Cells	BPSD Association
Cholinergic	Microglial α7nAChR	Activation → ↑ Aβ phagocytosis, ↓ TNF-α/IL-6, ↑ IL-4/IL-10 [100,101]
	Cholinergic projections (nucleus basalis)	Deficiency → psychosis, apathy, agitation [103]
	Cholinergic–glial α7nAChR (Nrf2-HO1)	Loss of cholinergic input removes anti-inflammatory brake → microglial inflammation [106,107]
	Astrocytic choline metabolism	Supports cholinergic function; its enhancement improves mood symptoms (e.g., with choline alphoscerate) [110]
	miR-98-5p (targets Chrna7)	Upregulated → ↓ α7nAChR → ↓synaptic proteins, ↑ NF-κB inflammation, ↓ Nrf2 antioxidants [114]
Monoaminergic	Microglial TLR4	Activation → ↑ TNF-α → ↓ brain 5-HT → depressive behavior [82]
	Microglial p38-MAPK/SERT	Cytokines alter SERT function → serotonergic dysfunction (depression/apathy) [47]
	Microglial IDO	Depletes tryptophan → ↓ 5-HT synthesis → depression, apathy [118]
	Astrocytic 5-HT_2A_ (striatum)	Reduced density → impaired 5-HT_2A_-dopamine crosstalk, ↓ dopamine release (early AD) [119]
	Astrocytic 5-HT_4_ receptor	Activates α-secretase → ↑ sAPPα, ↓ Aβ secretion [122,123]
	Astrocytic/microglial 5-HT_6_	Antagonism inactivates glia → reduces Aβ formation [124]
	Astrocytic DRD2/CRYAB	Loss of DRD2 signaling → ↑ IL-1β, IL-6 → neuroinflammation [118]
Glutamate/GABA	Astrocytic GLT-1	AD: ↓ glutamate reuptake, ↓ GluN2B → impaired E/I balance [132]
	Astrocytic glutamine synthesis	↓Glutamine → ↓ neuronal GABA → E/I imbalance → agitation, anxiety, sleep disturbances [133]
	Astrocytic mGluR5 dynamics	Biphasic changes (↑ at 7 months) coincide with hyperactivity [136]
	Astrocytic GAT3	↑ GAT3/benzodiazepine receptor density in temporal cortex → more severe depression [138]
	Astrocytic ketogenesis	Inhibits extrasynaptic NMDA receptors → anti-excitotoxic effect [141,142,143]

Notes: Arrows indicate the direction of effects: ↑ represents an increase or upregulation; ↓ represents a decrease or downregulation; → represents a causal relationship (leads to/results in).

**Table 3:** Glia-based therapeutics for BPSD.

Therapeutic Strategy	Mechanism	Intervention	Preclinical/Clinical Evidence
Microglial Anti-Inflammatory	promotes M2 polarization, reduces IL-1β/IL-6/TNF-α	CX3CL1-CX3CR1 axis modulators	Preclinical: alleviates stress-induced depressive-like behaviors [42]
	blocks IL-1β-mediated NMDAR hyperactivation, reverses repetitive behaviors	NLRP3 inhibitors (MCC950, dapansutrile) [226]	Preclinical: reversed by IL-1 receptor blockade or memantine [199]
	STING inhibition → reduces microglial synaptic engulfment and gliosis	STING inhibitor H-151	Preclinical: reduces gliosis in AD mice [198]
	Kv1.3 blockade	Kv1.3 blockade	Preclinical: conditional knockout reverses stress-induced Aβ deposition and cognitive deficits [162]
	blocks ATP-induced inflammasome activation and IL-1β release	JNJ-54175446 (selective brain-penetrant P2X7 antagonist)	Preclinical: attenuates anhedonia in rodent models; Clinical: safe/well-tolerated in MDD, blunted BzATP-stimulated IL-1β release [224]
	increases short-chain fatty acids → suppresses M1 polarization via MAPK	GV-971 (sodium oligomannate)	Preclinical: remodels gut microbiota, increases propionate/butyrate [225]
	Inhibition of microglial cytokine release and NLRP3/NF-κB pathways	Atypical antipsychotics (clozapine, quetiapine)	Clinical: exert part of BPSD efficacy [222,223]
	CSF1R inhibition	EI-1071	Clinical: Phase II (NCT06745583)
	CD3 molecule on T-cells	Nasal foralumab [227]	Clinical: Phase II (NCT06489548)
Astrocyte Homeostasis Restoration	Modulation of astrocyte glutamate clearance and synaptic remodeling	Clozapine	Preclinical: modulates astrocyte glutamate handling [228]
	Upregulation of astrocytic GDNF/BDNF release and connexin 43-mediated gap junction communication	Fluoxetine, amitriptyline	Preclinical: exert BPSD benefits [229,230,231]
	Modulation of astrocyte-centric BDNF-TrkB signaling	Ketamine	Preclinical: relevant to depression and AD [155,232]
	AMPK/CREB/BDNF signaling	Urolithin A (natural metabolite)	Preclinical: alleviates depression-/anxiety-like behaviors [233]
	Increase astrocyte glutamate uptake and glutathione levels; reduce NF-κB-mediated inflammation	Risperidone	Preclinical: reduces inflammation [234,235,236,237]
	Sustain astrocyte homeostasis during antipsychotic use	Resveratrol (adjuvant)	Preclinical: sustains homeostasis [222]
	Normalize hippocampal glutamate levels; reduce anxiety-like behaviors	Memantine	Preclinical: normalizes glutamate, reduces anxiety [139]
	Chemogenetic activation of hippocampal astrocytes	Chemogenetic activation (astrocytes)	Preclinical: in 5xFAD mice rescues LTP, memory, BPSD-like deficits, reduces Aβ plaque load [183]
Oligodendrocyte Myelin Repair	Promote oligodendrocyte differentiation and remyelination; increase mature oligodendrocytes via H3K9me3 epigenetic regulation	Clemastine (antimuscarinic)	Preclinical: rescues social avoidance and prefrontal hypomyelination in social isolation-induced depressive-like mice [76]
	BDNF protects OPCs from Aβ_1–42_-induced death and promotes proliferation/differentiation	BDNF	Preclinical: in vitro protection of OPCs [238]
	40 Hz gamma light/sound stimulation preserves white matter integrity	Spectris™ (Cognito Therapeutics)	Clinical: open-label extension reduced decline in emotional stability/social engagement (total scores –0.59 vs. −15.12 for sham, *p* = 0.0006) [238]
Glial–Neuronal Crosstalk Modulation	Astrocyte-mediated hippocampal glutamate regulation	Memantine (NMDA receptor antagonist)	Preclinical: improves BPSD-like behaviors (anxiety, depression) in AD mice [139]
	restores astrocyte metabolic support (glycolysis/lactate) via AhR	PF06840003 (brain-penetrant IDO1 inhibitor)	Preclinical: restores hippocampal glucose metabolism and behavioral deficits in AD models [239]
	p75 neurotrophin receptor modulation → reduces synaptic degeneration and glial abnormalities	LM11A-31	Preclinical: reduces BPSD-like deficits in mouse models; Clinical: consistent Phase 2a results [240]
	TREM2 agonism + riluzole (nanotherapeutic) → enhances microglial Aβ clearance, reduces glutamate accumulation	R@AClipo (COG1410 + riluzole)	Preclinical: mitigates agitation and sleep disturbances in AD mice [136]
Gene and Cell Therapies	Patient-derived iPSCs for individualized BPSD modeling	iPSCs with hindbrain organoids	Preclinical: hindbrain organoids from AD patients show patient-specific SSRI responses [241]
	GRP transplantation → stimulates endogenous neurogenesis/gliogenesis	GRP transplantation	Preclinical: into AD rats reverses anxiety-like and depressive-like behaviors, alters plasticity-related proteomes [242]
	CRISPR/Cas9 editing of TREM2 loss-of-function variants	CRISPR/Cas9	Preclinical: in human iPSC-derived microglia restores phagocytosis and reduces pro-inflammatory cytokines [243]
	Hematopoietic cell transplantation replaces Trem2-deficient microglia with wild-type myeloid cells	Hematopoietic cell transplantation	Preclinical: restores Aβ clearance and mitigates BPSD-like deficits in AD mice [244,245]
Emerging Agents for BPSD	suppresses microglial TNF-α, enhances BDNF via 5-HT_2A_/AhR, inhibits ROS/NO (5-HT_2_ receptor-dependent)	Psilocin (psilocybin metabolite)	Clinical: NCT04123314 (ongoing)—psilocybin (15/25 mg) for depression in MCI/early AD [246,247]
	reduces astrogliosis; indirect glial modulation via 5-HT_1A_	5-MeO-DMT (sublingual)	Clinical: NCT06812221 (completed Phase I/II) for anxiety/depression in mild-to-moderate AD [247]
	5-HT_1A_-mediated indirect glial modulation (astrocytes/microglia express 5-HT receptors, 5-HT_1A_ activation reduces neuroinflammation)	GH001 (inhaled 5-MeO-DMT)	Clinical: Phase 2b in TRD (*n* = 81)—MADRS reduction −15.5 vs. placebo (*p* < 0.001, d = −2.0), 57.5% remission at day 8, HAM-A reduction −10.0, no severe AEs
	Acts on CB2, 5-HT_1A_, PPARγ → anti-inflammatory, anxiolytic	CBD	Clinical: NCT04075435 (open-label, high-CBD/low-THC for anxiety/agitation in MCI or mild-to-moderate AD); NCT04436081 (RCT, crossover, THC-free CBD oil for agitation in AD)

## Data Availability

No new data were created or analyzed in this study. Data sharing is not applicable to this article.

## Abbreviations

The following abbreviations are used in this manuscript:

BPSD	Behavioral and psychological symptoms of dementia
AD	Alzheimer’s disease
MCI	mild cognitive impairment
Aβ	amyloid-beta
ADNC	Alzheimer’s disease neuropathologic change
MBI	Mild Behavioral Impairment
HR	Hazard Ratios
MTHFR	methylenetetrahydrofolate reductase
DAM	disease-associated microglia
CSF	cerebrospinal fluid
sTREM2	soluble TREM2
LDAM	lipid-droplet-accumulating microglia
NPS	neuropsychiatric symptoms
WMH	white matter hyperintensities
OR	odds ratio
CNS	Central Nervous System
AβOs	amyloid-β oligomers
TLR4	Toll-like receptor 4
5-HT	serotonin
IL-1ra	IL-1 receptor antagonist
GDNF	glial cell line-derived neurotrophic factor
LCN2	lipocalin-2
TBARS	Thiobarbituric Acid Reactive Substances
ACC	Anterior Cingulate Cortex
ChEIs	acetylcholinesterase inhibitors
α7nAChRs	α7 nicotinic acetylcholine receptors
SERT	serotonin transporter
IDO	indoleamine-2,3-dioxygenase
DRD2	dopamine D2 receptor
CRYAB	αB-crystallin
mGluR5	metabotropic glutamate receptor 5
CIH	chronic intermittent hypoxia
LRP	Lewy-related pathology
HMGB1	high-mobility group box 1
UCP4	uncoupling protein 4
eNMDARs	extrasynaptic NMDA receptors
COPD	Chronic Obstructive Pulmonary Disease
OPCs	oligodendrocyte progenitor cells
βHB	β-hydroxybutyrate
NPI	Neuropsychiatric Inventory
DAMPs	damage-associated molecular patterns
CAT	catalase
SSRIs	selective serotonin reuptake inhibitors
iPSCs	induced pluripotent stem cells
GRP	Glial-restricted precursor
CBD	Cannabidiol
MADRS	Montgomery–Åsberg Depression Rating Scale
HAM-A	Hamilton Anxiety Rating Scale
